# COVID-19 Pandemic Impact on Academic Global Health Programs: Results of a Large International Survey

**DOI:** 10.5334/aogh.3843

**Published:** 2022-09-29

**Authors:** Elizabeth S. Rose, Tracy L. Rabin, Jenny Samaan, James C. Hudspeth, Layan Ibrahim, Maria Catalina Padilla Azain, Jessica Evert, Quentin Eichbaum

**Affiliations:** 1 Vanderbilt University Medical Center, Vanderbilt University School of Medicine, US; 2 Department of Internal Medicine, Yale School of Medicine, US; 3 Child Family Health International, US; 4 Boston University, US; 5 Vanderbilt University School of Medicine, US; 6 Child Family Health International, University of California San Francisco, US

**Keywords:** Covid-19, academic global health programs, LMICs and HICs, virtual learning, international travel, risk mitigation

## Abstract

**Background:**

The COVID-19 pandemic caused significant disruptions in international communications and travel for academic global health programs (AGHPs) in both high-income countries (HICs) and low- and middle-income countries (LMICs). Given the importance of international travel and communication to AGHPs, the pandemic has likely had considerable impact on the education, research, and administrative components of these programs. To date, no substantive study has determined the impacts of the COVID-19 pandemic on AGHPs in HICs and LMICs. This study assessed the impacts and resultant adaptations of AGHPs to pandemic realities with the goal of sharing strategies and approaches.

**Methods:**

This study applied a mixed methods sequential explanatory design to survey AGHPs in HICs and LMICs about the impacts of the COVID-19 pandemic on three program domains: education, research, and administration. First, we surveyed a range of AGHP stakeholders to capture quantitative data on the pandemic’s impact. Subsequently we conducted semi-structured interviews with select survey participants to gather qualitative data expanding on specific survey responses. Data from both phases were then compared and interpreted together to develop conclusions and suggest adaptive/innovative approaches for AGHPs.

**Results:**

AGHPs in both HICs and LMICs were significantly impacted by the pandemic in all three domains, though in different ways. While education initiatives managed to adapt by pivoting towards virtual learning, research programs were impacted more negatively by the disruptions in communication and international travel. The impact of the pandemic on scholarly output as well as on funding for education and research was quite variable, although LMIC programs were more negatively impacted. Administratively, AGHPs implemented a range of safety and risk mitigation strategies and showed a low risk tolerance for international travel. The pandemic posed many challenges but also revealed opportunities for AGHPs.

**Conclusions:**

The COVID-19 pandemic disrupted AGHPs in HICs and LMICs in expected and unexpected ways. Programs noted some unanticipated reductions in education program funding, negative impacts on research programs, and reduced scholarly output. Many programs reported well-coordinated adaptive responses to the pandemic including, for instance, virtual (in place of in-person) collaboration in research. The pandemic will likely have lasting impacts with regard to education, research collaborations, and administration of programs.

## Introduction

The practice of global health relies on extensive international collaborations between institutions in high-income countries (HICs) and low- and middle-income countries (LMICs). In academia, such collaborations involve research and education as a component of university certificate, degree, and diploma programs. These programs often entail travel between HICs and LMICs to learn and conduct research. To effectively run such programs also requires administrators.

The term academic global health program (AGHP) refers to a spectrum of university-based initiatives that may include both education and research components and may be incorporated into undergraduate or postgraduate studies or within health professions schools. Although global health practitioners and partners have contributed to substantial improvements in public health systems and healthcare delivery and outcomes among vulnerable populations around the world [1,2], there also have been criticisms of poorly designed programs that circumvent and detract from local healthcare and health professions training systems. Increased awareness about issues of equity and colonialism as well as the pause on international travel precipitated by the pandemic have prompted practitioners to reflect on aligning intent and ethics of global health activities [3]. Global health academics are currently engaged in self-reflection about its historical roots and complicity in colonialism [4].

AGHPs in HICs often develop partnerships with institutions in LMICs that include trainee and faculty exchanges between institutions. These exchanges remain mostly unidirectional with trainees and faculty from HIC programs traveling to LMICs which points to the resource and power imbalances between HIC and LMIC programs. Such exchanges are usually administered and funded by HIC programs and governmental grants [5]. Given the colonial roots of global health, AGHPs in HICs are usually more embracive of the notion of global health and they are more numerous and better resourced than programs in LMICs.

The COVID-19 pandemic has had a major impact on the global health discipline in both HICs and LMICs, disrupting delivery of educational materials, development and implementation of research, and administration of program operations. Funding has often been cut, decreased, and/or siphoned away from global health programs [6]. Some programs managed to pivot and respond quite agilely, some scaled back operations, and others were compelled to shut down [7]. Research and educational exchanges of trainees and faculty between HICs and LMICs were halted as was international travel.

AGHPs have been reported to be negatively impacted by the pandemic, especially those with face-to-face classroom settings [3,8,9]. During the pandemic, many AGHPs adopted virtual learning approaches that required internet access. However, online learning presents challenges that vary between and within countries [10], including computer literacy [11], technology skills and access limitations [12], complications of different time zones for synchronous meetings [13], and, in areas with no or low internet bandwidth, inability to move programs online [12]. Despite challenges, virtual learning platforms have been used effectively within specific learning communities to raise awareness about public health issues, mobilize resources, and provide support without in-person contact [14].

With roots in tropical medicine, global health research has mostly entailed infectious disease and epidemiologic field research [4]. Often, research has been conducted in LMICs with funding and researchers from HICs, demanding frequent travel from HICs to LMICs. With severe disruptions in international travel during the pandemic, such research in AGHPs has been impeded and funding stopped, put on hold, or moved to other university programs [15]. Challenges to research during the pandemic included inability to meet in-person to conduct and discuss research; compromised research proposal review processes; and challenges to establishing collaborative networking at conferences [13].

Global health research programs initiated and funded in HICs are serviced by administrative offices that oversee grants, legal, safety, risk mitigation, and other operational and travel issues. AGHP administrative offices have been affected by the pandemic with some programs downsizing and others reportedly closing [9].

The pandemic impacted the research, education, and administrative arms of AGHPs. However, to date there has been no international survey to understand better how AGHPs worldwide have been impacted, have responded and adapted, and may emerge and change practices as the pandemic ebbs and flows. The aim of this study was to better understand the impact of the COVID-19 pandemic on AGHPs regarding (1) education, (2) research, and (3) administrative program challenges and opportunities, and to distill lessons learned into guidance for AGHP leaders and stakeholders.

## Methods

This study employed a mixed-methods sequential explanatory design. We used international global health and medical education associations’ listservs to invite educators, researchers, and administrators associated with AGHPs located in mostly high-income countries to complete an online survey and/or participate in a 30- to 40-minute semi-structured virtual interview. Due to the widespread, general outreach of our recruitment method and the anonymity of the survey, there may have been a few survey responses from the same AGHP. The associations we contacted were the following: Association of Medical Education in Europe (AMEE), The Japan Society for Medical Education, Association of American Medical Colleges Visiting Student Learning Opportunities Global Network, Consortium of Universities for Global Health (CUGH), Association of Faculties of Medicine of Canada (AFMC), Korean Association of Medical Colleges (KAMC), Bellagio Global Health Education Initiative (BGHEI), and ECFMG GEMx program/AFREHealth. These associations represent the medical education and global health associations globally which the authors contacted and were given access to. We administered the survey and interviews in English. Inclusion criteria required identification as an educator, researcher, or administrator in an AGHP. The definition of AGHP, as stated in the Introduction, did not vary by country.

The survey was divided into four sections: demographics, educators, researchers, and administrators. Question types included nominal measures, binary measures, multiple choice questions, Likert scales, and comment boxes. All participants responded to questions in the first section, (demographics) and responded to questions in the section(s) that corresponded to their self-identified role(s) in their AGHP: educator, researcher, and administrator. In the education section, domains of inquiry included: educational content, delivery, funding and scholarly output. Research domains included: budget allocation, grant funding, scholarly output, and virtual collaboration. Administration included: program operations including travel and risk mitigation for clinical and non-clinical program participants.

We used descriptive statistics to analyze the data and categorized data based on role and country income designation. Income designations included HICs and LMICs, collapsing more nuanced World Bank categories (https://data.worldbank.org/country). Since participants received only questions that corresponded with their role(s), we converted the proportion of respondents for each answer to a percentage of the total participants who received that question.

Interviews were conducted to further explore insights that appeared in survey data analysis as well as to gain additional insights not captured in the survey. We built the interview guide upon initial review of the quantitative data. Two co-authors conducted semi-structured interviews via Zoom at a time that was convenient for the participant. Written notes were taken during interviews, and audio recordings were made with the permission of the participants so that we could refer to the raw data if questions arose during analysis. We analyzed interview notes using a loose thematic analysis approach.

Ethics approval was received from Vanderbilt University Medical Center, IRB #210010. All survey and interview participants consented to participate in the study.

## Results

We present results specific to each research question under the relevant headings. Results are reported as the number (n) followed by percentage (e.g., HIC n: %; LMIC n: %). Additionally, although the goal of the study is to examine AGHPs globally and not compare the responses from HICs and LMICs, contrasting these sets of data yielded important insights and provided helpful perspectives. Therefore, we make such comparisons throughout the analysis while aware of the imbalance of the datasets (i.e., 85% of responses are from HICs) and limitations this poses on the validity of the comparisons.

## Phase 1 – Quantitative: Survey Analysis

### Demographics and Program Structures (Tables 1 and 2)

**Table 1:** List of countries by region with the number of countries responding from each global region (left column) and the number of respondents responding from each country (right columns).


REGION (*N* OF COUNTRIES)	COUNTRY (INCOME DESIGNATION)	RESPONDENTS (*N*)	

		SURVEY	INTERVIEW

**North America (2)**	United States of America (HIC)	161	10

	Canada (HIC)	7	

**Asia (8)**	India (LMIC)	3	

	Indonesia (LMIC)	1	

	Japan (HIC)	15	1

	Malaysia (LMIC)	2	

	Mongolia (LMIC)	1	

	Nepal (LMIC)	1	

	Pakistan (LMIC)	1	

	Vietnam (LMIC)	1	

**Latin America and the Caribbean (7)**	Antigua West Indies (HIC)	1	

	Brazil (LMIC)	11	

	Chile (HIC)	1	

	Costa Rica (LMIC)	1	

	Ecuador (LMIC)	1	

	Grenada (LMIC)	2	

	Guatemala (LMIC)	1	

**Europe (6)**	Germany (HIC)	3	

	Netherlands (HIC)	2	

	Romania (HIC)	1	

	Spain (HIC)	1	

	Sweden (HIC)	1	

	United Kingdom (HIC)	1	

**Middle East & North Africa (5)**	Egypt (LMIC)		1

	Israel (HIC)		1

	Oman (HIC)	1	

	Qatar (HIC)		1

	Syria (LMIC)	1	

**Sub-Saharan Africa (7)**	Democratic Republic of the Congo (LMIC)		1

	Ethiopia (LMIC)		1

	Kenya (LMIC)	2	

	Malawi (LMIC)	2	

	Mauritius (LMIC)	1	

	Nigeria (LMIC)	1	

	South Africa (LMIC)		1

**Oceania (1)**	Australia (HIC)	1	

**Total (36)**		**230**	**17**


HIC = high-income country. LMIC = low- or middle-income country. Country’s income designations were defined by the World Bank (https://data.worldbank.org/country).

**Table 2:** Demographics and Program Structures.


DEMOGRAPHIC CATEGORY	HIC N (%)	LMIC N (%)

Institution location (percent of total respondents)	196 (85%)	34 (15%)

Features of global health education programs		

Graduate	109 (56%)	24 (71%)

Undergraduate	61 (31%)	18 (53%)

Credit-bearing	147 (75%)	14 (41%)

Non-credit-bearing	123 (63%)	13 (38%)

International learning or research opportunities	150 (77%)	20 (59%)

Domestic learning or research opportunities (pre-pandemic)	169 (86%)	28 (82%)

New domestic learning or research opportunities established during pandemic	76 (39%)	7 (21%)


Demographic data obtained from the survey presenting features of global health education programs in high-income countries (HICs) and low- and middle-income countries (LMICs). HIC = high-income country. LMIC = low- and middle-income countries.

Survey respondents and interviewees represented 36 countries across seven geographical regions (Table 1). A total of 230 representatives of AGHPs responded to the survey. Of these, 196 (85%) respondents worked at institutions in HICs and 34 (15%) worked at institutions in LMICs (Table 2). For the qualitative portion, 29% of interviewees were from LMICs. Most respondents identified as global health educators (HIC 138: 70%, LMIC 14: 41%). Smaller numbers identified as global health researchers (HIC 70: 36%; LMIC 8: 24%) and administrators (HIC 89: 45%, LMIC 9: 26%). Programs across HICs and LMICs hosted both undergraduate and graduate courses in global health, for credit and not for credit.

Before the pandemic, more AGHPs in HICs than LMICs (77% vs. 59%) offered their trainees international learning or research opportunities.

### Education (Table 3)

**Table 3:** EDUCATORS: Key findings disaggregated by country income group.


EDUCATORS	HIC N (%)	LMIC N (%)
	
	138 (70%)	14 (41%)

Responses of global health education programs were		

Coordinated well or moderately well	107 (77%)	8 (57%)

Inadequately coordinated or uncoordinated	31 (23%)	5 (36%)

Changes in global health education programs as a result of the pandemic		

Changed delivery methods for education	109 (79%)	10 (71%)

Implemented different ways of engaging with partners	95 (69%)	2 (14%)

Changed some educational content	78 (57%)	8 (57%)

Modified requirements for program completion	41 (30%)	5 (36%)

Suspended outgoing travel	127 (92%)	3 (21%)

Suspended incoming visitors	91 (66%)	3 (21%)

Prior to the pandemic, programs used distance learning for curricular delivery		

Almost always	3 (2%)	2 (14%)

Often	23 (17%)	0 (0%)

Sometimes	59 (43%)	2 (14%)

Rarely or never	52 (38%)	7 (50%)

Don’t know	1 (1%)	1 (7%)

Impact of pandemic on global health education funding (internal/institutional)		

Moderately increased	3 (2%)	0 (0%)

No change	41 (30%)	4 (29%)

Moderately reduced	20 (14%)	0 (0%)

Significantly reduced	35 (25%)	2 (14%)

Don’t know/Other	37 (27%)	6 (43%)

Impact of pandemic on global health research funding (external)		

Significantly increased		

Moderately increased	4 (3%)	0 (0%)

No change	22 (16%)	0 (0%)

Moderately reduced	11 (8%)	0 (0%)

Significantly reduced	13 (9%)	1 (7%)

Other	5 (4%)	0 (0%)

Don’t know	30 (22%)	4 (29%)

Don’t have external funding for education	51 (37%)	7 (50%)

Impact on one’s scholarly output		

Considerably increased	5 (4%)	0 (0%)

Moderately increased	24 (17%)	1 (7%)

No change	39 (28%)	1 (7%)

Moderately decreased	40 (29%)	3 (21%)

Considerably decreased	27 (20%)	6 (43%)

Don’t know	1 (1%)	1 (7%)


HIC = high-income country. LMIC = low- and middle-income countries.

#### Program coordination

Most educators in HICs (107; 77%) stated that their program’s response to the pandemic was well-coordinated or moderately well-coordinated, compared with educators in LMICs (8: 57%). Educators who reported their program’s responses as inadequately coordinated or uncoordinated (HIC 31: 23%, LMIC 5: 36%) attributed such observations to their program’s lack of central coordination or leadership support, or to cancellations of classroom activities. In HICs, major changes in education programs included: changing education delivery methods (109: 79%) and implementing different ways of engaging with partners (95: 69%). In LMICs, major changes were made in education delivery methods (10: 71%) and educational content (8: 57%).

#### Distance learning

Educators reported that prior to the pandemic, their programs sometimes (HIC 59: 43%, LMIC 2: 14%) or rarely/never (HIC 52: 38%, LMIC 7: 50%) used distance learning for curricular delivery. Educators were varied in their plans to continue online education post-pandemic. Some educators hoped to return to in-person trainings, while others planned to continue online training.

#### Funding

Educators’ responses about the impact of the pandemic on institutional funding allocations varied. For some there was no change in funding (HIC 41: 30%, LMIC 4: 29%). HIC educators reported moderately (20: 14%) or significantly (35: 25%) reduced funding whereas LMIC educators were mostly not sure about changes in funding (6: 43%).

#### Scholarly output

Regarding scholarly output, most educators reported output had been moderately decreased (HIC 40: 29%, LMIC 3: 21%). Similar percentages in HICs reported no change (HIC 39: 28%, LMIC 1: 7%), while few reported that output was moderately (HIC 24: 17%, LMIC 1: 7%) or considerably (HIC 5: 4%, LMIC 0: 0%) increased.

#### Travel

Educators in HICs reported a high level of outgoing travel (92%) and incoming visitor (66%) suspension as well as implementing different ways of engaging with international partners (69%). In LMICs, only 21% suspended outgoing and/or incoming travel, and 14% implemented different ways to engage.

### Research (Table 4)

**Table 4:** RESEARCHERS: Key findings disaggregated by country income group.


RESEARCHERS	HIC N (%)	LMIC N (%)
	
	70 (36%)	8 (24%)

Impact of pandemic on global health research programs		

Very significant	24 (34%)	6 (75%)

Moderately significant	29 (41%)	1 (13%)

Small impact	9 (13%)	0 (0%)

No impact/Don’t know	6 (8%)	1 (13%)

Impact of pandemic on global health research funding (internal/institutional)		

Significantly increased	1 (1%)	0 (0%)

Moderately increased	1 (1%)	0 (0%)

No change	25 (36%)	0 (0%)

Moderately reduced	8 (11%)	1 (13%)

Significantly reduced	11 (16%)	5 (63%)

Don’t know/Other	22 (31%)	2 (25%)

Impact of pandemic on global health research funding (external)		

Significantly increased	1 (1%)	1 (13%)

Moderately increased	3 (4%)	0 (0%)

No change	20 (29%)	0 (0%)

Moderately reduced	7 (10%)	1 (13%)

Significantly reduced	9 (13%)	1 (13%)

The focus has shifted	6 (9%)	1 (13%)

Don’t know/Other	22 (32%)	2 (25%)

Impact on one’s scholarly output		

Considerably increased	3 (4%)	1 (13%)

Moderately increased	17 (24%)	1 (13%)

No change	14 (20%)	1 (13%)

Moderately decreased	18 (26%)	0 (0%)

Considerably decreased	13 (19%)	4 (50%)

Don’t know	3 (4%)	1 (13%)

Virtual collaboration could replace in-person aspects of global health research		

Entirely	4 (6%)	2 (25%)

Somewhat	49 (70%)	3 (38%)

Rarely or never	12 (17%)	3 (38%)

Don’t know	3 (4%)	0 (0%)


HIC = high-income country. LMIC = low- and middle-income countries.

#### Impact on research

Seventy-five percent of researchers in LMICs but only 34% of HIC researchers reported that the pandemic had a very significant impact on global health research programs at their institutions whereas more researchers in HICs reported moderately significant impact (HIC 29: 41%, LMIC 1: 13%).

#### Funding

In HICs, 29% of researchers reported no change in external funding and 32% were unsure of funding changes. Similarly, 25% of researchers in LMICs were unsure of changes. Regarding institutional funding, 16% of researchers in HICs reported that the pandemic had significantly reduced funding and about two-thirds of researchers reported no change (36%) or unsure of the impact on funding (31%). In contrast, in 63% of researchers in LMICs reported that the pandemic had significantly reduced such funding.

#### Scholarly output

Regarding scholarly output, similar percentages of researchers in HICs and LMICs reported no change (HIC 14: 20%, LMIC 1: 13%), but a higher percentage of researchers in LMICs (50%) than in HICs (19%) reported considerably decreased scholarly output.

#### Virtual collaboration

Many researchers suggested that virtual collaboration could entirely (HIC 4: 6%, LMIC 2: 25%) or somewhat (HIC 49: 70%, LMIC 3: 38%) replace in-person global health research collaborations. However, others did not believe virtual replacement to be feasible (HIC 12: 17%; LMIC 3: 38%).

### Administration (Table 5)

**Table 5:** ADMINISTRATORS. Key findings disaggregated by country income group.


ADMINISTRATORS	HIC N (%)	LMIC N (%)
	
	89 (45%)	9 (26%)

Program’s risk tolerance for re-initiating clinical/non-clinical global health experiences		

Low risk tolerance	58 (65%)/54 (61%)	7 (78%)/7 (78%)

Medium risk tolerance	26 (29%)/30 (34%)	0 (0%)/1 (11%)

High risk tolerance	5 (6%)/5 (6%)	1 (11%)/0 (0%)

Program’s decision structure to resume travel		

Centralized process	67 (75%)	7 (78%)

Decentralized process	7 (8%)	0 (0%)

No process	4 (4%)	1 (11%)

Unsure of process	14 (16%)	1 (11%)

Program’s decision criteria for resuming international exchanges		

Official international or national authority travel notices and advisories acceptable within institutional travel policies (e.g., WHO, CDC, etc.)	76 (85%)	8 (89%)

An approved vaccine is available to trainees/faculty	57 (64%)	6 (67%)

Destination country has acceptably low COVID-19 incidence rate	61 (69%)	6 (67%)

Destination country has adequate public health resources to manage cases and care for individuals who may contract COVID-19 (including personal protective equipment for clinical providers)	54 (61%)	5 (56%)

Assurance that travel routes and layovers meet adequate safety criteria	47 (53%)	5 (56%)

Partner institution official policies are in place and foreign visiting trainees are welcomed	58 (65%)	5 (56%)

Emergency medical, security, and travel insurance services are in place to provide evacuation support in the event of a COVID-19 outbreak	52 (58%)	6 (67%)

Assurance that laws are in place to protect stakeholders from litigation if COVID-19 transmission happens while students/faculty are participating in global health activities	32 (36%)	5 (56%)

Not yet determined	12 (15%)	0 (0%)

Other	2 (2%)	0 (0%)

Program’s decision criteria for allowing visiting faculty and trainees		

Official international or national authority travel notices and advisories acceptable within institutional travel policies (e.g., WHO, CDC, etc.)	61 (69%)	8 (89%)

Visiting trainees/faculty have proof of vaccination	39 (44%)	7 (78%)

Visiting trainees/faculty have a negative test at the time of arrival	56 (63%)	7 (78%)

Assurance that travel routes and layovers meet adequate safety criteria	29 (33%)	6 (67%)

Confidence that the countries or regions students/faculties come from have adequate testing and have emerged from the pandemic	31 (35%)	6 (67%)

Our institution has adequate medical resources to manage cases of COVID-19, if identified on campus	37 (38%)	5 (56%)

Hosted international visitors have evidence of adequate insurance to cover illness or evacuation in the event of becoming infected	45 (46%)	7 (78%)

Host community has a low COVID-19 incidence rate	26 (27%)	5 (56%)

Not yet determined	24 (27%)	0 (0%)

Other	3 (3%)	0 (0%)


HIC = high-income country LMIC = low- and middle-income countries.

#### Risk tolerance and travel

In determining criteria for sending trainees and faculty abroad, most program administrators noted that their institution had a low-risk tolerance for re-initiating clinical (HIC 58: 65%, LMIC 7: 78%) and non-clinical global health experiences (HICs 54: 61%, LMIC 7: 78%). Programs in both HICs and LMICs had a low-risk tolerance for these experiences.

Similar percentages of respondents in HICs and LMICs (around 67%) suggested that the decision for international travel to resume would rest on an approved vaccine being available; the destination country having acceptable low COVID-19 incidence rate; and/or the destination country having adequate public health resources to manage cases. The decision to allow faculty and trainees from other countries to visit followed an analogous pattern (proof of vaccination; negative test on arrival; safe travel routes and layovers).

## Phase 2 – Qualitative: Thematic Analysis of Interviews

We received 23 responses expressing an interest in being interviewed (10% of the number who responded to the survey). From these, we secured interviews with 17 individuals of whom 4 (29%) were from LMICs, reflecting a similar HIC:LMIC ratio of respondents as in the survey. However, no individuals from the South American or European regions expressed interested in being interviewed.

We present here a loose thematic analysis of the interview data (including direct quotes from interviewees) – first the general responses of interviewees are presented, and then those from their self-identified program roles: education, research, administration.

### General response to impact of the pandemic

As in the survey, most interviewees reflected that their program’s response to the pandemic was coordinated moderately well or well. Key components of such coordination revealed in the interviews included resources for online teaching, program flexibility, proactive leadership, and effective communication. Communication was most often described using words such as “clear” and “transparent.” Less positive responses about coordination included that that leadership was “slow in making decisions” and “unclear about travel regulations” and that “decisions seemed to come out of nowhere.”

### Education – Impact on virtual learning programs

All interviewees described a shift to virtual learning that was motivated by a desire to continue students’ successful progression through the program. As a result of moving education online, most interviewees reported few, if any, lapses in educational continuity. As in the quantitative survey, most interviewees in both HICs and LMICs predicted a hybrid model would likely be adopted. Interviewees did express concerns about online platforms pertaining to accreditation of programs and difficulties in getting hands-on training for future health professionals.

An insight not revealed in the survey but expressed in both HIC and LMIC interviews was how crucial and helpful information technology (IT) departments had been during the pandemic. However, some programs in LMICs found the shift to online learning difficult due to lack of internet connectivity and access to computers. One LMIC interviewee stated, “We do not plan to do virtual teaching when we don’t have to. We have no funds to give students laptops, so that is an issue.”

### Research – Scholarly output

Interviewees described a range of ways that their research was impacted. A LMIC interviewee commented that, “Every corner of research was affected… We lacked reviewers who could log into the online platforms used.” Most interviewees agreed that online platforms would be incorporated to some extent in future research collaborations although drawbacks to online collaborations were reported, including limited social interaction, poor internet connectivity, lack of necessary tools and resources, and limited conference networking to find collaborators.

For clinician researchers, the increased burden of clinical work during the pandemic severely detracted from scholarly output. Other interviewees suggested that their scholarly output fluctuated and initially improved when the pandemic subsided over the summer of 2021 but worsened with the ensuing Delta surge. Two interviewees remarked that bench research was also impacted, especially prior to availability of the vaccine when most researchers were compelled to stay home.

One researcher remarked that it was too early to assess the impact on scholarly output since many articles were “backlogged for peer review and acceptance into journals” due to the increased number of submissions during the pandemic. Moreover, some published articles “bypassed peer review” and this presented a “challenge to academic scholarship standards.” One interviewee suggested that scholarly output was improved due to researchers “having more time working from home.”

### Funding – Impact on global health programs

As in the survey, interviewees’ responses to questions about funding were quite varied although responses appeared somewhat more positive than in the survey. Most interviewees suggested the pandemic had little to no impact on internal institutional funding for global health programs. Several remarked that funding shifted from global health projects to those directly associated with the pandemic. Some programs reported receiving additional institutional support for innovative programs, including funding for tutoring and mentoring of nurses working in resource poor communities.

As in the survey, several interviewees in HICs corroborated that funding for travel to LMICs was lost. Some programs lost institutional funding for student travel grants, while others will be able to carry over funds to a new fiscal year, thereby increasing future support for students. During the pandemic one participant temporarily lost an external global health scholarship but was informed it would be “re-instituted” when the pandemic was resolved but the interviewee “did not let the funding changes impact my own research… I went ahead and used my own financial resources.”

Less travel also created more time for data analysis and project innovation and leftover funds were diverted to research. In cases where researchers already had established collaborations with whom they could communicate via online platforms, scholarly output was less impacted by funding.

### Administrators – Risk tolerance and safety

All except one HIC interviewee reported a low risk level for international travel. One HIC program reported not allowing any travel even within the country. Some programs that disallowed international student travel, focused on local (“glocal”) health initiatives in local low resource settings or pivoted to remote internships. LMIC programs were generally more risk tolerant than HIC programs. Reflecting the survey data, most programs did not report a higher risk tolerance between clinical and non-clinical travel requests.

Programs with medium tolerance based their risk evaluation on the following criteria: vaccination status of their students/faculty and host partners, adherence to safety protocols, use of PPE and masks, COVID-19 testing availability, and institutional committees to evaluate travel requests. Two interviewees reported changing risk tolerance based on the decreased number of COVID-19 cases in the country.

### Open, unstructured reflections

At the conclusion of the interviews, interviewees were asked if there was anything else about the impact of the COVID-19 pandemic on their global health program they would like to share. The responses were categorized into positive (14) and negative (8) responses.

Positive impacts included new and creative uses of technology, dedication and commitment of global health staff, pivots and changes in the curriculum, program alignment with learning objectives, and new opportunities provided during the pandemic. Additionally, shifting priorities included embracing global and local elective courses and program focus, committing to international partners, and increasing focus on decolonizing global health.

Negative impacts included the deleterious effect on mental health of health care workers and the overall stress and strain on all personnel. Some reflected on the loss of colleagues to COVID-19. Others reflected on the impact on students, including that first-year medical students missed the opportunity to bond with classmates. At one institution, applications to their medical school had declined due to COVID-19 and the restrictions placed on international travel. In reviewing interview data, it was difficult to distinguish if these impacts were specific to the AGHP or institution wide.

## Discussion

The geographical representation of survey responses was extensive. Most respondents reported that the pandemic had a significant impact on their programs with respect to education, research, and administration. Of interest, almost a third more HIC than LMIC programs reported a higher level of coordination. These differences may have reflected relative differences between HICs and LMICs in available resources and capacities to respond to an emergency like the pandemic.

The level of coordination applied especially to the rapid switch toward online learning platforms. Considering that around 40% of respondents reported rarely or sometimes using remote learning prior to the pandemic but now many are using remote learning frequently, and given the paucity of evidence-based literature to guide remote learning, the rapid adaptation to this mode of learning points to remarkable institutional adaptiveness, resilience, and learning agility [16,17]. Such adaptiveness and agility may be derived from experiences working in the resource limited settings of global health (also termed “resourcefulness learning” [18]).

Respondents remarked that, despite some shortcomings, they were impressed by the benefits of online learning and planned to retain it in some form post-pandemic. This finding aligns with findings of other published studies that showed how programs effectively alternated synchronous and asynchronous online learning and supplemented it with other online messaging applications [13,19]. Given the extensive use of online platforms and other messaging and applications, it is not surprising that the biggest impact of the pandemic in education was the method of educational delivery (71%) rather than content, although more than half of respondents also reported content changes. As reported by Krohn et al. [20], prior to the pandemic many AGHP faculty lacked experience with online delivery technologies and new learning modalities [20]. Changes in educational content may have been driven by constraints with online delivery methods and perceived need to change content to have relevance to the pandemic.

It is noteworthy that a quarter of researchers in the survey reported that virtual collaboration could entirely replace in-person collaboration, and some commented that they had more frequent meetings online during the pandemic than in-person pre-pandemic. While an increased frequency of online meetings is understandable during a pandemic, what deserves further investigation is how virtual collaboration could completely replace in-person field work. Virtual collaboration may entail little field work with research design and data analyses performed online. If in-person collaboration is considered less vital, the re-aligned role of the HIC research partner also comes into question. Shifts to virtual collaboration raises the potential of reallocating funding previously used by HIC researchers to travel to LMIC research sites. The reallocation of funds, which could include moving extra funding to LMIC institutions, reducing funding since research travel costs are lower, or allocating extra funding to other global health research or education projects. These changes could have significant implications on global health research and programs.

If shifts towards more virtual aspects of research come to fruition, there may be continued shifts towards increased scholarly outputs due to increased time available to write (articles, grants) that was previously dedicated to travel, although this shift will likely be more prevalent among HIC researchers who historically have been the primary travelers in global health collaborations. Overall, it was unsurprising that the impact of the pandemic was more pronounced in the domain of field research than on education given the reliance of global health field research on international travel. Other studies have similarly reported on the, mostly negative, impacts the pandemic placed on global health education and research [15,21].

Regarding scholarly output, three-quarters of researchers in LMICs and a third of those in HICs reported that the pandemic had a very significant, presumably negative, impact on their global health field research and scholarly output. The more significant impact on LMIC research programs may be associated with their lack of local funding and resources and a dependence on funding from HICs.

The impact of the pandemic on funding of AGHPs was varied and did not follow a pattern. Some reported increased institutional funding (some of which was retrieved from unused travel funds) to develop innovations, research, or establish new programs, whereas others had funds diverted away from global health. Some respondents were understandably not aware of the changes in institutional funding levels as they were not privy to such information. Nonetheless, educators in LMICs were twice as likely as their HIC counterparts to be unsure of change in funding, a finding that may represent the power imbalance in which program funding in LMICs originates in HICs [22,23]. The fact that about 40% of AGHPs in HICs and more than 60% in LMICs experienced moderately or significantly reduced funding likely reflected differences in available and readily allocatable resources. However, a comment by a LMIC respondent that funding had significantly decreased as it depended on students visiting from HICs was unexpected.

In HICs, it was not surprising to learn that funding for travel was decreased or removed since it could not be utilized. In some cases, funding was carried over to the next fiscal year, suggesting that institutions may have predicted the pandemic to end sooner than it has. Such diversion of global health travel funds aligned with other published reports in which travel funds were diverted towards improving longitudinal global health programs and reduced global health travel was substantiated as a reduction in carbon footprints in the interests of planetary health [21].

A noteworthy difference between the concerns of HIC and LMIC programs to the pandemic (especially regarding research) was that HICs focused on international travel and partner engagement, whereas LMICs focused on their home programs and education/research content and delivery. This focus of HIC programs on travel reflected the predominantly unidirectional travel from HICs to LMICs in global health. However, one might have expected travel restrictions to have had a more significant negative impact on scholarly output in HIC programs. Thus, it was surprising to find that almost one-quarter of researchers reported increased scholarly output. Such increased output could possibly be attributed to the halt on travel that freed up time to write and produce scholarship.

Regarding risk tolerance, HIC program administrators were three times as likely as their LMIC counterparts to report suspending incoming and/or outgoing travel. This finding reflected further the unidirectional nature of global health travel from HICs to LMICs, with LMIC institutions hosting HIC visitors. LMIC institutions may not have felt empowered to halt such incoming travel, while HIC institutions felt they had the power to suspend travel. Furthermore, the power differential between HICs and LIMCs was evident as HIC program administrators were twice as likely as LMIC counterparts to report that they had not determined criteria for incoming visitors but had for outgoing travel, thus appearing to prioritize sending their trainees and faculty abroad rather than receiving international visitors. However, it is also possible that travel decisions for some programs were made at the institutional level, beyond the control of the AGHP.

### Limitations of Study

The study had several limitations. The number of AGHPs in HICs in the world is unknown but high, and our responses represent only a small fraction. Given the low number of LMIC respondents, it is unclear how representative these data are, and the LMIC information could reasonably be regarded as hypothesis generating. This low number is unsurprising, as there are not many established AGHPs in LMICs. A disproportionate number of the study’s AGHPs were in the United States of America (USA) (82%), the survey was conducted in English, and the authors are all from USA-based institutions. This structural imbalance between HIC and LMIC AGHPs could not have been rectified by sampling or survey distribution methods. The statistical analyses were descriptive as the skewed ratio of respondents precluded correlational and higher level of statistical analyses. Nonetheless, the data provided helpful insights into the impacts of the pandemic and adaptive trends in AGHPs in HICs and LMICs.

This study was conducted only in English and may have missed input from other major language groups. The survey was sent to organizations such as the Association for Medical Education in Europe, Japan Society for Medical Education, and Korean Association of Medical Colleges, whose members’ primary language is not English. However, given the general ubiquity of English in global health, the survey would likely have been accessible to most, although specific nuances may have been missed.

The sequential explanatory study design entailed a cross-sectional survey conducted over a period of two months followed three months later by interviews conducted over another period of two months. The dynamics and ground situation of the fast-moving pandemic were constantly changing during and between the survey and the interview periods. Hence, the views and responses of study subjects may have changed over time.

The thematic analyses of the interviews were based on notes written during the interviews, which may present biases and a limitation of the study [24]. However, given that the comments of the 17 interviewees were quite disparate, it was not clear how amenable they would have been to coding analysis. Transcription of audio recording is costly and time-consuming [25] and may not be necessary depending on the type of analysis and level of granularity required [26,27]. For the purposes of this study’s sequential explanatory design, written notes provided adequate explanations to the survey findings. Interviewee responses were illuminating and provided explanations that were not captured in the survey, thus substantiating application of the mixed methods sequential explanatory study design.

## Conclusion

While some of the findings in this study might have been predicted, others were unexpected and are worthy of further comment. An unexpected finding was the immense adaptiveness, learning agility, and innovation that AGHPs displayed in dealing with the pandemic. AGHPs developed a deeper realization of their interconnectedness and a revitalized willingness to learn from one another.

There were similarities and differences in the ways that AGHPs were impacted and dealt with the pandemic. The most notable impact on HICs was travel restrictions. The most notable impact on LMICs was associated with lack of resources and reduction or loss of funding for projects. The differences in the impact of the pandemic on AGHPs in HICS and LMICs could mostly be ascribed to differences in resources between countries, established power differentials entrenched global health practices, and funding of collaborative projects that are controlled largely by HICs.

### Way forward?

The rapid and relatively easy adoption of virtual learning platforms and modalities that AGHPs accomplished (and grew to appreciate) suggested that online learning will remain in some form as part of AGHPs and will continue to be improved.

The gradual realization by AGHPs that intensive travel is not critical for sustaining programs suggested that global health travel may be substantially reduced post-pandemic. This change will likely be supported by the global movement toward “decolonizing” global health [22,23,28] but a reduced in-country presence will not fully address the need to shift power imbalances that are prevalent in global health collaborations. It is also likely that new students entering the field of global health will be more involved in local low-resource health settings (“glocal”) rather than undertaking international travel for field work in LMICs.

The global health field will likely be transformed considerably by the time the pandemic abates. This transformation will be a consequence of the impact of the pandemic as well as of the transformative movement to “decolonize” the discipline. The value of studies, such as this one, is to gain pre-emptive understanding on how the field may change and to suggest ways of adapting accordingly.

## Additional Files

The additional files for this article can be found as follows:

10.5334/aogh.3843.s1 Supplementary 1. Electronic Survey.

10.5334/aogh.3843.s2 Supplementary 2. Semi-structured Interview Guide.
