# Supplementary material for: COVID-19 Pandemic Impact on Academic Global Health Programs: Results of a Large International Survey
Source: Ann Glob Health. 2022 Sep 29;88(1):84. doi: 10.5334/aogh.3843 (PMC9524235; doi:10.5334/aogh.3843)
Supplement: Supplementary 1. — Electronic Survey. [file agh-88-1-3843-s1.pdf]

COVID-19 Pandemic - Impact on academic global health programs

The purpose of this study is to evaluate the impact of the COVID-19 pandemic on global health education and research programs at academic institutions located mostly in high-income countries (HICs). The results of the survey will provide an understanding of how programs in different countries have coped with education, research, and administrative issues related to the pandemic, and how they envisage their path forward as the pandemic resolves.

The survey below should take no more than 5 - 7 minutes to complete. Your participation is voluntary and all responses are confidential and will be maintained in a password-protected database. Only de-identified collated information will be shared at meetings or in medical journals.

If you are unable or unwilling to participate, please forward this survey to colleagues at your institution, and to others in your country who may be willing to participate.

If you have any questions, please contact Quentin Eichbaum at quentin.eichbaum@vumc.org.

We would be grateful if you could submit your responses by March 21, 2021.

Thank you for your time and consideration. We greatly appreciate your willingness to help us all better understand the impact of the COVID-19 pandemic on global health programs.

Sincerely

Dr Quentin Eichbaum

Dr Jessica Evert

Dr James Hudspeth

Dr Tracy Rabin

Dr Elizabeth Rose

Dr Jenny Samaan

Do you agree to participate in this study?

◯ Yes
◯ No

Demographics and Program Information

Are you affiliated with an academic institution / university?

(Note: This refers to the place in which you conduct most of your global health education, training, or research activities.)

◯ Yes
◯ No

My institution is...

◯ Public
◯ Private

Please write the country where your institution is located.

____

What kinds of global health programs or experiences does your institution offer? [please select all that apply]

☐ Undergraduate global health DEGREE program (e.g. Bachelors)
☐ Graduate/postgraduate DEGREE program (e.g. Doctorate, Masters, Honors)
☐ For credit: Minors, certificates, scholarly concentrations, clinical tracks, electives
☐ Not for credit: Volunteer/extracurricular experiences (e.g. mission trips, service learning)
☐ Other - please specify
☐ None

Please describe "other"

____

Please answer the following questions regarding the Global Health Education and/or Research programs you are involved with at your institution.

Before the COVID-19 pandemic did these global health program(s) offer your own students/trainees learning or research opportunities in other countries?	○ Yes, in low- and middle-income countries<br>○ Yes, in high-income countries<br>○ Yes, in low-, middle-, and high-income countries<br>○ No
Before the COVID-19 pandemic, did your global health program(s) offer your own students/trainees domestic learning or research opportunities within your country?	○ Yes<br>○ No
As a result of COVID-19, have you established or expanded domestic global health opportunities within your country (e.g. at rural/low resource sites)?	○ Yes<br>○ No
Which of the following best describes your role(s) in the global health program(s) at your institution?<br><br>If you have more than one role, please select ALL that apply.	☐ Global health educator<br>☐ Global health researcher<br>☐ Administrator, director, risk manager, or legal officer<br>☐ None

Questions for Global Health Educators

My institution's response to the COVID-19 pandemic with respect to our global health education program(s) has/have been...

○ Well-coordinated
○ Moderately well-coordinated
○ Inadequately coordinated
○ Uncoordinated

Please explain your answer choice.

____

In response to the COVID-19 pandemic our global health education program has... [please select all that apply]

- ☐ Changed some educational content
- ☐ Changed delivery methods for education
- ☐ Changed requirements for program completion
- ☐ Suspended outgoing international travel
- ☐ Suspended incoming international travel
- ☐ Implemented different ways of engaging with international partners
- ☐ I don't know/not sure
- ☐ Other(s)

Please explain "other" __________________________________

PRIOR TO the COVID-19 pandemic, our global health EDUCATION programs...

- ◯ Rarely or never used distance learning for curricular delivery
- ◯ Sometimes used distance learning for curricular delivery
- ◯ Often used distance learning for curricular delivery
- ◯ Almost exclusively used distance learning for curricular delivery
- ◯ I don't know/not sure

SINCE the start of the COVID-19 pandemic, our global health program...

- ◯ Rarely or never uses distance learning for curricular delivery
- ◯ Sometimes uses distance learning for curricular delivery
- ◯ Often uses distance learning for curricular delivery
- ◯ Almost exclusively uses distance learning for curricular delivery
- ◯ I don't know/not sure

Describe change(s) that your global health education program(s) have implemented since the pandemic started for trainees at your institution. __________________________________

If applicable, describe change(s) in your global health education program(s) that have been implemented since the pandemic started for trainees at your partner institutions. __________________________________

Given your program's experience with distance learning to date, are there changes you expect to make in your global health education programs once the COVID-19 pandemic recedes? Please explain. __________________________________

What impact has the COVID-19 pandemic had on internal/institutional budget allocation for your global health education programs?

- ◯ Funding has been significantly reduced
- ◯ Funding has been moderately reduced
- ◯ There has been no reduction in funding
- ◯ Funding has been moderately increased
- ◯ Funding has been significantly increased
- ◯ I don't know/not sure
- ◯ Other

Please explain "other" __________________________________

To what extent has external grant funding for your global health education programs been affected by the COVID-19 pandemic?

- ◯ We do not have grant funding for global health education
- ◯ Funding has been significantly reduced
- ◯ Funding has been moderately reduced
- ◯ There has been no reduction in funding
- ◯ Funding has been moderately increased
- ◯ Funding has been significantly increased
- ◯ I don't know/not sure
- ◯ Other

Please explain "other" __________________________________

To what extent has your own scholarly activity (e.g. publications, conferences, abstracts) related to global health education been affected by the pandemic?

- ◯ Considerably reduced
- ◯ Moderately reduced
- ◯ No change
- ◯ Moderately increased
- ◯ Considerably increased
- ◯ I don't know/not sure

Please provide any additional comments regarding your experiences about the impact of the COVID-19 pandemic on global health education at your institution and/or at partner/training sites abroad. __________________________________

Questions for Global Health Researchers

What overall impact has the COVID-19 pandemic had on global health research programs at your institution?

- ◯ Very significant impact
- ◯ Moderately significant impact
- ◯ Small impact
- ◯ No noticeable impact
- ◯ I don't know/not sure

Please describe any significant impacts. __________________________________

What impact has the COVID-19 pandemic had on internal/institutional budget allocation for your global health research?

- ◯ Funding has been significantly reduced
- ◯ Funding has been moderately reduced
- ◯ There has been no reduction in funding
- ◯ Funding has been moderately increased
- ◯ Funding has been significantly increased
- ◯ I don't know/not sure
- ◯ Other

Please explain "other" __________________________________

To what extent has external grant funding for your global health research been affected by the COVID-19 pandemic?

◯ Funding has been significantly reduced
◯ Funding has been moderately reduced
◯ There has been no reduction in funding
◯ Funding has been moderately increased
◯ Funding has been significantly increased
◯ The funding focus has shifted
◯ I don't know/not sure
◯ Other

Please explain "other"

____

To what extent has your scholarly activity (e.g. publications, conferences, abstracts) related to global health research been affected by the pandemic?

◯ Considerably reduced
◯ Moderately reduced
◯ No change
◯ Moderately increased
◯ Considerably increased
◯ I don't know/not sure

Based on your experience before and during the COVID-19 pandemic, how do you think virtual collaboration might replace in-person aspects of global health research?

◯ Virtual collaboration can entirely replace in-person aspects
◯ Virtual collaboration can somewhat replace in-person aspects
◯ Virtual collaboration can rarely replace in-person aspects
◯ Virtual collaboration can never replace in-person aspects
◯ I don't know/not sure

Describe one (or more) change(s) that you have implemented in your global health research program since the pandemic started.

____

Please provide any additional comments regarding your experiences about the impact of the COVID-19 pandemic on global health research at your institution and/or at partner sites abroad.

____

Questions for Administrators, Directors, Risk Managers, and Legal Officers

How will your institution decide when international global health travel can be resumed for trainees and faculty? [please select all that apply]

☐ Our institution's risk management office will make/participate in that decision.
☐ Our institution's legal office will make/participate in that decision.
☐ Our institution has a committee that will make a recommendation to senior leadership who will ultimately decide.
☐ This is a decentralized decision and individual departments/units will decide for their staff.
☐ We have no process in place yet for when we will allow international travel.
☐ I am unsure how that decision will be made.
☐ Other

Please explain "other"

____

What is your institution's risk tolerance for re-initiating NON-CLINICAL global health education or research experiences (for students, scholars, or faculty) in other countries as the pandemic subsides?

Low risk tolerance: We are unlikely to support travel abroad until the situation is back to near normal (e.g. low infectivity/death rates, vaccine program initiated)

Medium risk tolerance: If the trainee accepts the risk and the respective governments allow travel, we will support an international experience provided appropriate pre-departure risk mitigation training is completed, and all appropriate levels of approval are met.

High risk tolerance: If the trainee accepts the risk and the respective governments allow travel, we will support an international experience and assume the trainee/faculty will take all necessary precautions.

◯ Low risk tolerance
◯ Medium risk tolerance
◯ High risk tolerance

What is your institution's risk tolerance for re-initiating CLINICAL global health education or research experiences (for students, scholars, or faculty) in other countries as the pandemic subsides?

Low risk tolerance: We are unlikely to support travel abroad until the situation is back to near normal (e.g. low infectivity/death rates, vaccine program initiated)

Medium risk tolerance: If the trainee accepts the risk and the respective governments allow travel, we will support an international experience provided appropriate pre-departure risk mitigation training is completed, and all appropriate levels of approval are met.

High risk tolerance: If the trainee accepts the risk and the respective governments allow travel, we will support an international experience and assume the trainee/faculty will take all necessary precautions.

◯ Low risk tolerance
◯ Medium risk tolerance
◯ High risk tolerance

Which of the following criteria do you think your institution will need to have in place before allowing trainees and faculty to go abroad? [please select all that you think might apply]

☐ Official international or national authority travel notices and advisories acceptable within institutional travel policies (e.g. WHO, CDC, etc.)
☐ An approved vaccine is available to trainees/faculty
☐ Destination country has acceptably low COVID-19 incidence rate
☐ Destination country has adequate public health resources to manage cases and care for individuals who may contract COVID-19 (including personal protective equipment for clinical providers)
☐ Assurance that travel routes and layovers meet adequate safety criteria
☐ Partner institution official policies are in place and foreign visiting trainees are welcomed
☐ Emergency medical, security, and travel insurance services are in place to provide evacuation support in the event of a COVID-19 outbreak
☐ Assurance that laws are in place to protect stakeholders from litigation if COVID-19 transmission happens while students/faculty are participating in global health activities
☐ It has not yet been determined / I have no idea
☐ Other

Please explain "other"

____

Which of the following criteria do you think your institution will need to have in place before allowing foreign trainees/faculty to VISIT your institution? [please select all that in your opinion might apply]

- ☐ Official international or national authority travel notices and advisories acceptable within institutional travel policies. (e.g. WHO, CDC, etc.)
- ☐ Visiting trainees/faculty have proof of vaccination
- ☐ Visiting trainees/faculty have a negative test at the time of arrival
- ☐ Assurance that travel routes and layovers meet adequate safety criteria
- ☐ Confidence that the countries or regions students/faculties come from have adequate testing and have emerged from the pandemic
- ☐ Our institution has adequate medical resources to manage cases of COVID-19, if identified on campus
- ☐ Hosted international visitors have evidence of adequate insurance to cover illness or evacuation in the event of becoming infected
- ☐ Host community has a low COVID-19 incidence rate
- ☐ It has not yet been determined / I have no idea
- ☐ Other

Please explain "other"

____

Please share any additional information regarding the ways in which your institution has responded and/or will respond to the COVID-19 pandemic.

____

Final questions

Please provide any comments on the impact of COVID-19 on your academic global health programs if there are areas we did not address.

What is the name of your institution? (optional)

____

If you would like to receive the results of this survey upon publication, a link to opt in by providing your email address will be available when you click "submit."
