# Supplementary material for: COVID-19 Pandemic Impact on Academic Global Health Programs: Results of a Large International Survey
Source: Ann Glob Health. 2022 Sep 29;88(1):84. doi: 10.5334/aogh.3843 (PMC9524235; doi:10.5334/aogh.3843)
Supplement: Supplementary 2. — Semi-structured Interview Guide. [file agh-88-1-3843-s2.pdf]

Supplement 2: Semi-structured Interview Guide

Interview Guide:

Suggested Text to Be Read to Respondents Who Have Agreed to Be Interviewed

DEAR [INTERVIEWEE'S NAME]
Thank you again for agreeing to take part in this interview. My name is Dr Quentin Eichbaum and I am a trained researcher conducting this study. We are conducting this interview to gather more information about the perceived impact of the COVID-19 impact on education programs and research related to global health.
For the duration of the interview, I will ask questions about your experience at your institution related to global health education program and research during the past year. The interview should last about 30-40 minutes. I would also like to remind you that your participation in this interview is completely voluntary and can be stopped at any time.
I will be taking notes during the interview, but to ensure the integrity of the interview and that none of our conversation is missed, it would be most helpful to record the conversation, if you agree to it. The recording will be destroyed after the transcription of the recording has been made. Our discussion will remain strictly confidential and will only be accessed by the small research staff of this project.
Do you agree to recording? [If yes, begin recording]
Do you have any other questions before we begin?

Sample of interview Questions:

1. Please characterize your institution's response to the COVID pandemic on a scale of well-coordinated to not well-coordinated.

    Possible further probing questions:
    Why did you characterize your institution's response in that way?
    Please give specific examples of what your institution/program did.

2. Please describe your experience at your institution with virtual learning and education in global health programs during the pandemic.

    Possible further probing questions:
    Please explain how you anticipate online platforms being used in your global health programs post-pandemic.

3. Please describe your experience at your institution with using virtual platform for global health research collaborations during the pandemic.

    Possible further probing questions:
    Please explain the level to which virtual platforms could fully replace in person collaborations in research.

4. Please describe changes in internal and external funding levels at your institution or within your global health programs and research during the pandemic.

   Possible further probing questions:
   - Please explain why these changes occurred.
   - Please explain the impact of these funding changes on your global health programs and research.

5. Describe your institution's general risk tolerance for sending students/scholars abroad.

   Possible further probing questions:
   - if your institution has a higher risk tolerance for sending clinical for sending students/scholars abroad for clinical rotations than for non-clinical, could you explain the difference in risk tolerance.

6. *(For respondents who indicate in the email above that they are "Researchers").* Describe how external funding for global health research impacted scholarly output at your institution.

   Possible further probing questions:

   - If scholarly output was not impacted by levels of research funding, what explanation might you offer for this occurrence?
   - If scholarly output increased when research funding decreased, what factors might have contributed to this inverse relationship?

7. Is there anything else that you would like to share about the impact of the pandemic on your institution?

Closing text to interview:
Thank you for sharing your thoughts and experiences. I appreciate the time that you gave to this interview. If you have any questions following our conversation, you may contact me at ***quentin.eichbaum@vumc.org.*** We will share the study results with you once they are analysed and published. Thank you again!
